# Preferences and Willingness to Pay for Health App Assessments Among Health Care Stakeholders: Discrete Choice Experiment

**DOI:** 10.2196/57474

**Published:** 2025-05-26

**Authors:** Anna-Lena Frey, Simon Leigh, Carla Toro, Carme Pratdepàdua Bufill, Charles McCay, Tatjana Prenđa Trupec, Giuseppe D'Avenio, Menno Kok, Antanas Montvila, Philipp Goedecker, Petra Hoogendoorn

**Affiliations:** 1 Organisation for the Review of Care and Health Apps Daresbury United Kingdom; 2 Warwick Medical School University of Warwick Coventry United Kingdom; 3 Fundació TIC Salut Social Barcelona Spain; 4 Ramsey Systems Ltd Shrewsbury United Kingdom; 5 Care and Public Health Research Institute Maastricht University Maastricht The Netherlands; 6 National Center for Innovative Technologies in Public Health Istituto Superiore di Sanità Rome Italy; 7 EIT Health Belgium and The Netherlands Rotterdam The Netherlands; 8 Hospital of Lithuanian University of Health Sciences Kaunas Clinics Kaunas Lithuania; 9 COCIR (Comité de Coordination Européen de l’industrie radiologique, électromédicale et informatique des soins de santé) Brussels Belgium; 10 National eHealth Living Lab Department of Public Health and Primary Care Leiden University Medical Center Leiden The Netherlands

**Keywords:** health apps, quality assessment, discrete choice experiment, willingness to pay, health system, app developers

## Abstract

**Background:**

The adoption of high-quality health apps has been slow, despite the myriad benefits associated with their use. This is partly due to concerns regarding the effectiveness, safety, and data privacy of such apps. Quality assessments with robust and transparent criteria can address these concerns and, thereby, encourage the use of high-quality apps. However, a major challenge for such assessments is reaching a scale at which a substantial proportion of the more than 350,000 available health apps can be evaluated.

**Objective:**

To support the scaling of health app quality assessments, this study aimed to examine the preferences and willingness to pay for assessments with different value propositions among potential customers.

**Methods:**

We conducted 2 discrete choice experiments: one with 41 health app developers and another with 46 health system representatives (from health care institutions, authorities, and insurers) from across Europe. Mixed logit models were applied to examine the impact of assessment attributes on participants’ choices as well as to calculate marginal willingness to pay and predicted assessment uptake.

**Results:**

Among health app developers, the attributes with the largest impact on assessment choices were the associated clinical care uptake (integration into clinical guidelines and reimbursement or procurement) and cost (purchase price). Increased willingness to use assessed apps and app store integration of assessment results had a moderate impact on choices, while required developer time investment and time until assessment results become available made the smallest contribution. Among health system representatives, increased willingness of clinicians and patients to use evaluated apps had the greatest impact on assessment choices, followed by cost. Time until assessment result availability and the percentage of peers recommending the assessment made a moderate contribution, while reassessment frequency had the smallest impact on choices. On average, health app developers were willing to pay an additional €9020 (95% CI €4968-€13,072) if an assessment facilitates guideline integration and procurement or reimbursement (at the time of data collection, €1=US $1.11), while health system representatives were, on average, willing to pay €7037 (95% CI €4267-€9806) more if an assessment results in a large, rather than a small, increase in willingness to use the evaluated app. The predicted uptake of assessments that offer the preferred values for all attributes was 88.6% among app developers and 91.1% among health system representatives.

**Conclusions:**

These findings indicate that, to maximize uptake and willingness to pay among health app developers, it is advisable for assessments to facilitate or enable clinical guideline integration and reimbursement or procurement for high-scoring apps. Assessment scaling thus requires close collaboration with health authorities, health care institutions, and insurers. Furthermore, if health system organizations are targeted as customers, it is essential to provide evidence for the assessment’s impact on patients’ and clinicians’ willingness to use health apps.

## Introduction

### Background

There is increasing recognition that digital health technologies, including health apps that support health system services, disease prevention, self-management, and treatment, should play a greater role in health care [1]. Health apps complement in-person care and could ease the strain on clinicians and health systems, which is partly due to a shortage of health care professionals [2,3].

A number of recent studies have demonstrated the beneficial effects of health apps on health outcomes and quality of life [4], including in condition areas such as mental health [5,6], diabetes [7-9], hypertension [7], cancer [10,11], and asthma [12]. Moreover, health apps can contribute to cost savings and more efficient use of health system resources that are facing an increasing demand [13,14]. Indeed, past estimates suggest that using health apps to their full potential could have resulted in vast savings across the European Union in 2017 [15].

To attain these benefits, the widespread use of health apps is necessary, yet adoption has been slow [16], with concerns related to the effectiveness, safety, usability, and data privacy of such apps reported as a major barrier to their use among both patients and clinicians [17,18].

Importantly, these apprehensions are justified in some cases [19], and current European regulations are arguably not sufficient to fully alleviate such concerns [20,21]. First, the Medical Device Regulation (MDR) only covers apps that are *specifically intended by the developer (“manufacturer”) to be used for medical purposes*. This creates a “loophole” related to the phrasing of an app’s intended purpose, incentivizes development of apps with nonmedical purposes, and leaves a substantial number of health apps unregulated by the MDR [22]. For instance, in a sample of 271 health apps examined for the Dutch Ministry of Health, 215 apps (79.3%) did not fall under the MDR [23]. Such apps, as well as apps falling under MDR risk class I, are not required to undergo conformity assessment by a notified body [23]. Second, although the General Data Protection Regulation applies to health apps available in Europe that process personal data, compliance with this regulation is usually not independently evaluated. Indeed, it has been found that a large proportion of health apps on the market do not meet relevant data protection requirements [24,25]. Third, concerns have been raised regarding the quality and appropriateness of the studies that are deemed acceptable under the MDR to demonstrate a product’s safety and effectiveness [26,27]. Finally, the only user-facing output of the MDR assessment is a Conformité Européenne (CE; European Conformity) mark, which consists of the letters “CE” being affixed to medical devices, including health apps, if they have been found to comply with the MDR (through self-assessment for class I apps or through evaluation by a notified body for apps with a higher risk classification). This CE mark only aims to indicate conformity with the MDR, without seeking to provide transparency to users unfamiliar with regulatory requirements regarding the domains that have been assessed and how apps compare across them.

Partly as a result of the aforementioned considerations, a number of third-party health app assessment frameworks have been established by organizations such as health authorities, health care institutions, medical professional associations, and for-profit companies to promote the use of high-quality apps across Europe and beyond [28,29]. However, a major challenge for such assessment initiatives is reaching a scale at which they can evaluate a large proportion of the more than 350,000 health apps available on the global market [30] or even of the approximately 100,000 health apps available in Europe [29,31,32]. Such scaling can only be achieved if, alongside an efficient assessment process and a sufficient number of assessors, an appropriate customer group is targeted and customers’ expectations regarding assessment costs and benefits are met.

### Objectives

To facilitate the scaling of health app quality assessments, this study aimed to gather insights into the preferences and willingness to pay for health app assessments with different value propositions among potential assessment customers across Europe. For this purpose, employees of health app development companies and health system representatives (from health care institutions, health authorities, health technology assessment bodies, and health insurers) were invited to take part in a discrete choice experiment (DCE).

The study was conducted as part of the European Union–funded Label2Enable project, which aims to support the adoption and operation of the CEN ISO/TS 82304-2 health app quality assessment as a trusted standard across Europe [33,34]. However, the study’s findings are not specific to the CEN ISO/TS 82304-2 assessment and provide valuable information for the scaling of health app quality evaluation initiatives more generally.

## Methods

### Overview

DCEs are commonly used in health care contexts to examine stakeholders’ preferences [35]. In such experiments, participants are presented with several choice sets consisting of 2 or more alternatives (eg, 2 treatment options) that differ across a range of attributes (eg, cost, effectiveness, and side effects). Participants repeatedly choose between alternatives with different attribute values, allowing researchers to determine participants’ preferences as well as the trade-offs that participants are willing to make across attributes.

As part of this study, the DCE methodology was applied to elicit preferences for health app quality assessments with different value propositions from potential customers of third-party assessments. The study was designed and conducted in line with best practices developed by the International Society for Pharmacoeconomics and Outcomes Research Conjoint Analysis Working Group [36].

### Selection of Attributes and Levels

To identify relevant assessment attributes for the DCE as well as realistic values for these attributes, interviews were conducted with representatives of digital health assessment organizations from 7 European countries (for details, refer to Table S1 in Multimedia Appendix 1). The interviews, which also served to inform other aspects of the Label2Enable project, covered topics such as assessment scope, assessment trigger, time requirements, costs and funding, benefits of the assessment for health care stakeholders, progress to date, and barriers to scaling.

Subsequently, a video conference discussion was held with representatives of the stakeholder groups targeted by this study to determine the attributes they regarded as most important. Each representative was called on individually to comment on the attributes identified during the interviews and to suggest additional aspects to consider. All attributes that were indicated as being important by any of the representatives were shortlisted. More specifically, 2 shortlists were created (with some overlapping attributes): one for health app developers and another for health system representatives (from health care institutions, health authorities, health technology assessment bodies, and health insurers). Creating 2 attribute lists for use in 2 separate DCEs was deemed necessary due to the differing priorities and objectives of these stakeholder groups. For instance, an important attribute for health app developers was the amount of time they would need to invest to prepare the necessary documentation for the assessment. By contrast, this attribute was not relevant for health system representatives because they are not involved in gathering documentation as part of the assessment process.

**Figure 1:**
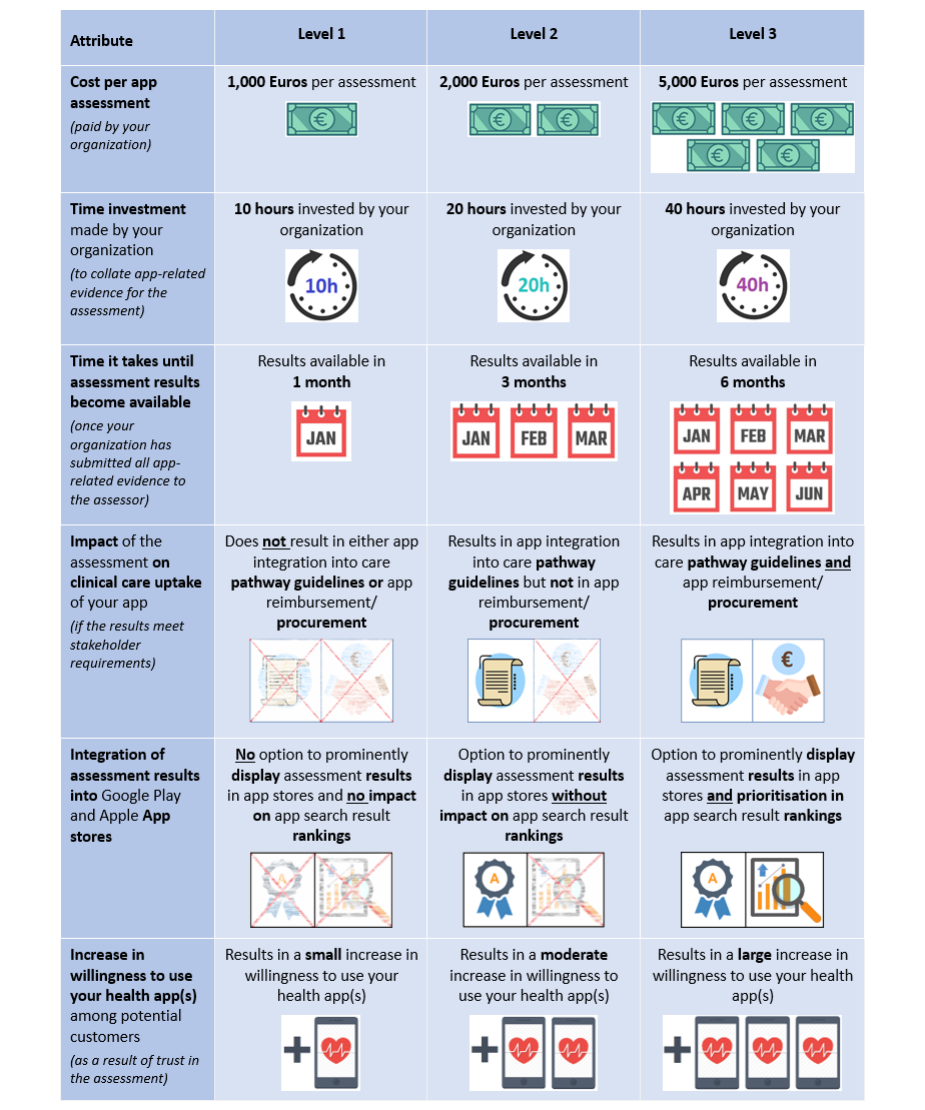
Attributes and levels presented to health app developers during the discrete choice experiment.

**Figure 2:**
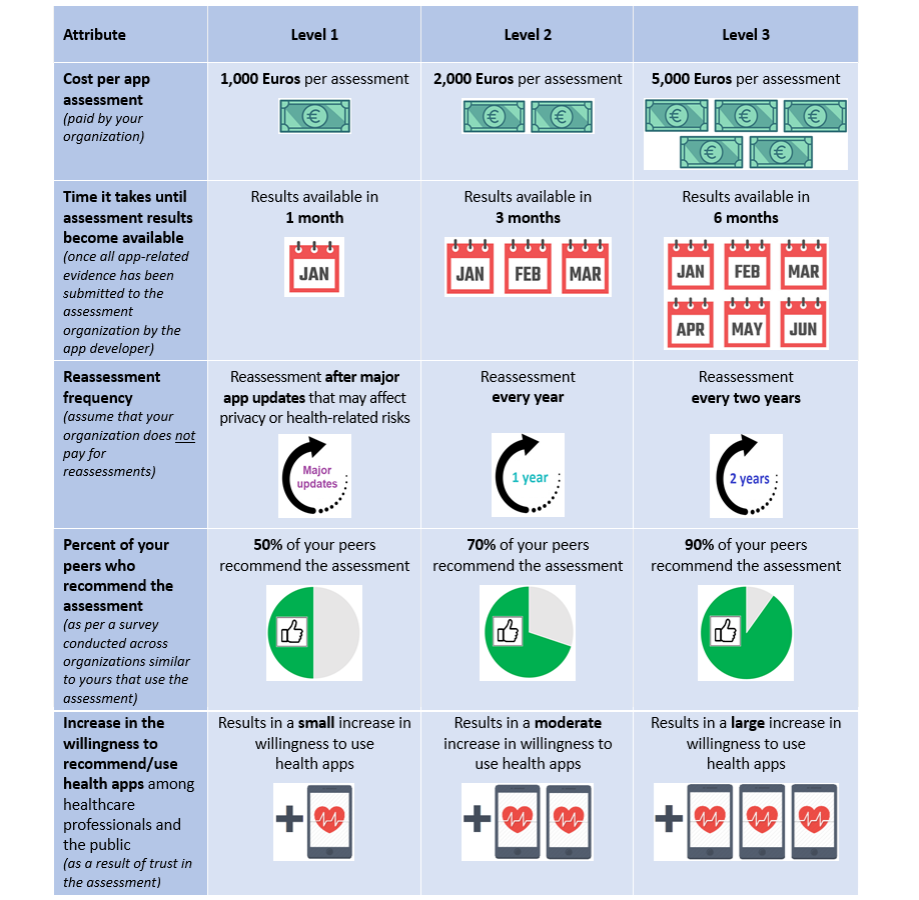
Attributes and levels presented to health system representatives during the discrete choice experiment.

The attribute shortlists were further refined through email correspondence with the stakeholder representatives until a consensus agreement on 5 to 6 attributes was reached. This number of attributes was chosen to limit the cognitive load of the choice task with full profiles. The levels for the selected attributes were based on the range of values reported by the assessment organizations during the interviews as well as the values observed during pilot testing of the CEN ISO/TS 82304-2 assessment. In line with recommendations [36], no more than 3 levels were chosen for each attribute. The final lists of attributes and levels for the 2 DCEs, as presented to participants, are shown in Figures 1 and 2.

### Choice Task

For the choice task, a forced-choice elicitation format with 2 alternatives was selected. Specifically, for each choice set, participants were presented with 2 assessments and asked to select the option that they would recommend to their organization. Other approaches, such as presenting >2 alternatives or requiring the ranking of a larger number of options, were deemed to be too cognitively demanding due to the number of included attributes.

After participants had made a selection for a given choice set, they were asked to choose between the assessment they had just selected and an opt-out option (ie, recommending that their organization does not make use of any assessment). Including an opt-out option is beneficial for determining demand [36]. However, if opt-outs are offered as a third option as part of the main choice set, the results can be difficult to interpret. This is because participants may select the opt-out option for reasons unrelated to a true preference, such as to minimize effort and avoid difficult choices [37,38]. The dual response design used in this study has been suggested as a way to address this issue because it requires participants to choose between the offered alternatives on each trial before being given the option to opt out [38].

To ensure that participants could reasonably evaluate choice sets that contained all selected attributes (ie, full profiles), a pilot test was conducted with 15 participants for both DCE versions. The pilot feedback indicated that participants did not find the choice task too complex; thus, full profiles were used.

### Experimental Design

The experimental design was generated using Ngene software (version 1.2; ChoiceMetrics Pty Ltd) [39]. For each of the 2 DCEs, a model was specified (refer to the Data Analysis subsection for details) and, through various iterations, the fractional factorial design with the lowest D-error value, and thus the highest statistical efficiency, was selected [39]. As recommended by the Ngene manual when no previous results are available [39], we used small priors, which indicated the expected sign of the parameters. Specifically, priors for cost, developer time investment, and assessment completion time were assigned a negative sign (−0.00001), and priors for the remaining benefit-related attributes were assigned a positive sign (0.00001).

Each DCE included 18 choice sets generated by Ngene, which, based on the pilot testing, was deemed to yield a suitable survey length without causing fatigue or dropout. In addition, a “rationality test” was incorporated, in which 1 assessment contained presumably preferable values for all attributes compared to the alternative option (ie, a lower level for the first 3 attributes and a higher level for the remaining attributes shown in Figures 1 and 2). The inclusion of such a test is recommended practice to determine whether participants demonstrate rational choice behavior, which is an assumption of the DCE methodology.

### Elicitation Method and Data Collection Instrument

The DCEs were presented to participants within a web-based Google Forms survey. At the start of the survey, the following background information was collected: the type, size, and country of the participants’ organization; the participants’ role in digital health–related decision-making; their previous experience with health app assessment; their views on whether making use of health app assessment would be beneficial for their organization; and their opinion on whether displaying assessment information should be mandatory or voluntary for health app developers.

Health system representatives were additionally asked to rank their preferences for different ways of engaging with health app assessments. Specifically, they ranked the following options from 1 (most preferred) to 5 (least preferred): paying for third-party assessment, paying for access to a library of assessed apps, performing assessments in-house, relying on self-assessments from health app developers, and not making use of assessments. The order in which these options appeared during the ranking task was randomized across participants.

After the background questions, DCE instructions, including a detailed description of each attribute level and an example choice set of 2 assessments, were presented. The DCE was framed by asking participants to imagine that their organization had selected a health app for potential submission to a third-party quality assessment and that they had been asked to make a recommendation regarding which assessment, if any, their organization should purchase. Choice sets were presented in table format, as shown in Figures 1 and 2 but with 2 alternatives.

### Participants

The study sample consisted of 41 employees of health app development companies (from hereon referred to as “health app developers” for short) and 46 health system representatives from across Europe. On the basis of power calculations, using the formula recommended by Johnson and Orme [40], which incorporates the number of attribute levels, choice sets, and alternative options, a sample size of 40 participants was required for each group.

The survey was promoted through newsletters of relevant professional organizations, such as the Healthcare Information and Management Systems Society, as well as through social media, professional networking sites, and contacts of the Label2Enable project team.

### Data Analysis

Responses to the background questions were summarized using descriptive statistics. For the rankings of assessment options provided by health system representatives, chi-square values were calculated as described by Finch [41]. Owing to a technical issue, information for 1 (2%) of the 41 health app developers was missing regarding their role in decision-making as well as their organization’s size and country.

Data from the 2 DCEs were analyzed using 2 separate mixed logit models, with observed assessment choices as the dependent variable and attribute levels of the presented assessments as predictors. In both models, cost (purchase price) and assessment completion time were entered as fixed, continuous variables to allow for the calculation of marginal rates of substitution without complications related to determining mean values [42]. All other attribute levels were entered as random, categorical variables, thus accounting for preference heterogeneity across participants. The categorical variables were dummy coded using level 1 (as indicated in Figures 1 and 2) as the reference category (refer to the paper by Daly et al [43] for a discussion of effects coding vs dummy coding for choice models).

The opt-out decisions, collected after the initial choice between assessments A and B, were incorporated into the analysis as recommended by Diener et al [44]. Specifically, trials on which participants selected the same assessment (A or B) for both the initial question (A vs B) and for the opt-out question (previously selected assessment [A or B] vs no assessment) were treated as 1 choice set with 3 options (A, B, and no assessment). By contrast, trials on which participants selected “no assessment” after initially choosing assessment A or B were treated as 2 choice sets with 2 (A and B) and 3 (A, B, and no assessment) options, respectively. In addition, an alternative specific constant (coded as 1 for “no assessment” and 0 for assessments A and B) was added to the model as a random effect.

Parameter estimation was conducted in Stata (version 18; StataCorp LLC) [45] using 2000 Halton draws to achieve stable coefficients. A sensitivity analysis was performed in which participants who did not select the expected option in the rationality test (health app developers: 3/41, 7%; health system representatives: 5/46, 11%) were excluded. The estimated coefficients for this analysis are presented in Multimedia Appendix 1. In the Results section, the estimates across all participants are reported. This is recommended practice, given that the deletion of *potentially* “irrational” responses may lead to the introduction of biases and the reduction of statistical efficiency and power [46].

Moreover, relative attribute importance was calculated by first subtracting the coefficient of the least preferred level from the coefficient of the most preferred level for each attribute and then dividing this range for a given attribute by the sum of these ranges across all attributes.

Mean marginal willingness to pay and marginal rates of substitution for assessment completion time were calculated by dividing the estimated model coefficients of the other attributes by the negative coefficients of cost and assessment completion time, respectively. SEs for these values were calculated using the delta method, as instantiated by the *nlcom* function in Stata.

In addition, predicted choice probabilities were examined for the best-, middle-, and worst-case scenarios, with all attributes set to the most preferred, middle, and least preferred levels, respectively, using the methodology and Stata code described by Lancsar et al [42].

Finally, a linear regression analysis was conducted to examine whether responses to the background questions predicted the percentage of times participants opted for “no assessment” during the DCE (refer to Multimedia Appendix 1 for the details and results of this analysis).

### Ethical Considerations

The study was reviewed and approved by the Biomedical and Scientific Research Ethics Committee of the University of Warwick (BSREC 82/22-23).

All participants provided informed consent at the start of the survey, after being presented with an information sheet outlining the purpose, methodology, and data-handling arrangements of the study. Participants could withdraw from the study at any point before submitting their responses, in which case none of the survey data were saved. All collected data were fully anonymous. No personally identifiable information was collected at any stage of the survey. Participants were not offered any compensation for completing the survey. This was made clear in the information sheet.

## Results

### Health App Developer Survey

#### Participant Characteristics

Health app developers were located across 18 European countries, including France (8/40, 20%), Germany (4/40, 10%), the Netherlands (4/40, 10%), the United Kingdom (4/40, 10%), Denmark (3/40, 8%), and Portugal (3/40, 8%; refer to Table S2 in Multimedia Appendix 1 for the full list of countries).

Most health app developers were based in micro (<10 employees; 11/40, 28%) or small (10-49 employees; 18/40, 45%) companies, rather than medium (50-249 employees; 6/40, 15%), large (250-999 employees; 2/40, 5%), or very large (≥1000 employees; 3/40, 8%) organizations.

Of the apps developed by the participants’ companies, 37% (15/41) provided operational support (eg, enabling web-based consultation with health care professionals or offering administrative services), 46% (19/41) supported disease prevention (eg, promoting a healthy lifestyle), 61% (25/41) facilitated self-management (eg, providing disease-specific information or symptom management aid), 66% (27/41) supported health care delivery (eg, assisting with diagnosis, clinical decision-making, treatment, or remote follow-up), 24% (10/41) offered health research support (eg, gathering patient-reported outcomes for clinical trials), and 5% (2/41) had other core functionalities not captured by the aforementioned categories.

With regard to digital health–related decisions, 18% (7/40) of the health app developers reported being the sole or final decision maker, 45% (18/40) indicated being part of a group of decision makers, and 35% (14/40) stated that they provided advice to inform decisions, while 2% (1/40) indicated having no influence on decision-making.

The majority of health app developers (32/41, 78%) had prior experience with health app assessment, which was indicated to include regulatory certification. Of these developers, 47% (15/32) reported that this experience had been mostly positive, while 34% (11/32) and 19% (6/32) stated that their experience had been neutral or mostly negative, respectively.

In addition, across all health app developers, 49% (20/41) strongly agreed and 44% (18/41) agreed that it would be advisable and beneficial for their organization to submit their health apps to a third-party quality assessment in the next 2 years, while 5% (2/41) and 2% (1/41) disagreed and strongly disagreed with this statement, respectively.

Finally, 59% (24/41) of the health app developers stated that, in their opinion, it would be preferable if it was *mandatory* for health app developers to display their app’s quality assessment results or a statement that their app has *not* been assessed in app stores and marketing materials, while the remaining 41% (17/41) thought such reporting should be *voluntary*.

#### Model Estimation Results and Relative Attribute Importance

The mixed logit model estimation results for the health app developer DCE are presented in Table 1. All attributes made a significant contribution to assessment choices, with coefficients associated with levels 2 and 3 differing significantly from those associated with the reference level 1 in all cases, except for the levels of “20 hours developer time investment” and “moderate increase in willingness to use assessed apps.”

The estimated coefficients for the sensitivity analysis, excluding participants who did not select the expected option in the rationality test, are presented in Table S3 in Multimedia Appendix 1.

All attribute levels had the expected sign and order, with health app developers, on average, preferring assessments that have a lower cost (purchase price), involve less developer time investment, make results available more quickly (ie, have a shorter assessment completion time), facilitate broader clinical care uptake via clinical guideline integration and procurement or reimbursement, offer full app store integration (ie, display of assessment scores and impact on search result rankings), and lead to a larger increase in willingness to use assessed apps.

Relative attribute importance values indicated that the largest contribution to health app developers’ choices was made by the clinical care uptake attribute (40.3%), followed by cost (17.9%). Increased willingness to use assessed apps (13.8%) and app store integration (13.6%) had a moderate impact on choices, while assessment completion time (9.5%) and developer time investment (4.8%) made the smallest contribution (refer to Figure S1 in Multimedia Appendix 1).

**Table 1:** Mixed logit model estimation results for health app developers.

		Mean coefficient, (SE)	*P* value	SD (SE)	*P* value
Cost (per €100)		−0.04 (0.01)	<.001	—^a^	—
Assessment completion time (per month)		−0.16 (0.03)	<.001	—	—
**Developer time investment (h)**					
	10 (reference)	0.00 (N/A^b^)	—	—	—
	20	−0.38 (0.23)	.10	0.52 (0.39)	.18
	40	−0.40 (0.19)	.04	0.61 (0.19)	<.001
**Impact on clinical care uptake**					
	None (reference)	0.00 (N/A)	—	—	—
	Guideline integration	1.23 (0.20)	<.001	0.63 (0.26)	.02
	Guideline integration and procurement or reimbursement	3.33 (0.56)	<.001	1.92 (0.42)	<.001
**App store integration**					
	None (reference)	0.00 (N/A)	—	—	—
	Assessment score display	0.46 (0.15)	.003	0.01 (0.11)	.92
	Assessment score display and impact on app search result rankings	1.12 (0.20)	<.001	0.74 (0.22)	<.001
**Increase in willingness to use assessed apps**					
	Small (reference)	0.00 (N/A)	—	—	—
	Moderate	0.35 (0.26)	.18	0.61 (0.37)	.10
	Large	1.14 (0.25)	<.001	0.93 (0.27)	<.001
Alternative specific constant for “no assessment”		0.25 (0.53)	.63	2.14 (0.52)	<.001

^a^Details for the SD (SE and *P* value) are not applicable to fixed effects.

^b^N/A: not applicable (SD, SE, and *P* values are not applicable to reference levels).

#### Marginal Willingness to Pay and Marginal Rates of Substitution for Assessment Completion Time

**Figure 3:**
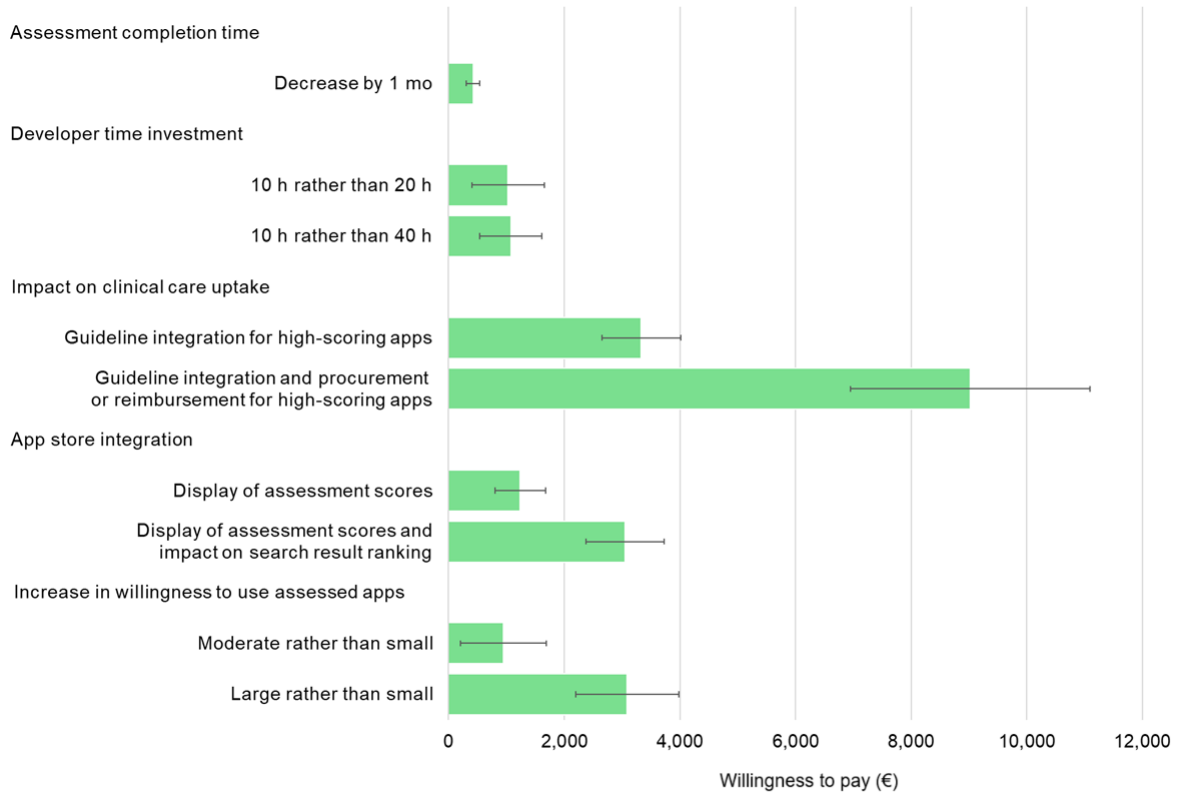
Health app developers’ marginal willingness to pay for different assessment attribute levels (error bars represent SEs). Note that the currency exchange rate at the time of data collection was approximately €1=US $1.11.

On average, health app developers were willing to pay an additional €9020 (95% CI €4968-€13,072) if an assessment facilitates guideline integration and procurement or reimbursement for high-scoring apps (ie, for apps that meet the requirements of stakeholders involved in clinical care uptake decisions). The marginal willingness to pay was only approximately a third of this amount if an assessment offers full app store integration (€3052, 95% CI €1722-€4381); or leads to a large, rather than a small, increase in willingness to use assessed apps (€3090, 95% CI €1341-€4840). Marginal willingness to pay for receiving the assessment results 1 month sooner was €425 (95% CI €196-€654; refer to Figure 3 and Table S4 in Multimedia Appendix 1 for the full results).

Moreover, health app developers were willing to wait an additional 21 months (95% CI 8-34 mo) for the evaluation results if the assessment facilitates clinical guideline integration and procurement or reimbursement for high-scoring apps. Furthermore, they were willing to wait approximately 7 months longer for assessment results if full app store integration (95% CI 3-11 mo) or a large, rather than small, increase in willingness to use assessed apps (95% CI 2-12 mo) is offered. For a cost reduction of €100, they were willing to wait an extra week (0.2 mo, 95% CI 0.1-0.4 mo) for the assessment results (refer to Table S5 in Multimedia Appendix 1 for all findings).

#### Predicted Assessment Uptake

Predicted assessment uptake was examined for the best-, middle-, and worst-case scenarios, with all attribute levels set to the most preferred, middle, and least preferred levels, respectively.

For the best-case scenario, the predicted assessment uptake among health app developers was 88.6% (95% CI 77.9%-96.5%) while for the middle- and worst-case scenarios, the predicted uptake was 49.2% (95% CI 38.9%-62.8%) and 12.4% (95% CI 4.8%-22.1%), respectively.

### Health System Representative Survey

#### Participant Characteristics

Health system representatives worked at the following types of organizations: public health care institutions (18/46, 39%), private health care institutions (10/46, 22%), health authorities (8/46, 17%), health technology assessment bodies (6/46, 13%), private health insurance companies (3/46, 7%), and public health insurance companies (1/46, 2%). These organizations were distributed across 13 European countries, including Spain (14/46, 30%), Belgium (5/46, 11%), the Netherlands (5/46, 11%), the United Kingdom (5/46, 11%), Croatia (4/46, 9%), and Lithuania (3/46, 7%; refer to Table S6 in Multimedia Appendix 1 for the full list of countries).

The organizations’ size ranged from very large (≥1000 employees; 15/46, 33%) and large (250-999 employees; 6/46, 13%) to medium (50-249 employees; 12/46, 26%), small (10-49 employees; 10/46, 22%), and micro (<10 employees; 3/46, 7%). The vast majority of organizations (41/46, 89%) were currently using, promoting, reimbursing, or otherwise interacting with digital health technologies.

Most health system representatives reported being part of a group of individuals making digital health–related decisions (25/46, 54%) or providing advice to inform such decisions (14/46, 30%), while 2% (1/46) indicated being the sole or final decision maker related to digital health, and 11% (6/46) stated that they did not contribute to digital health–related decisions at their organization.

The majority of health system representatives (34/46, 74%) had previous experience with health app assessment. Of these, 76% (26/34) reported that this experience had been mostly positive, while 21% (7/34) and 3% (1/34) indicated that their experience had been neutral or mostly negative, respectively.

Furthermore, of all health system representatives, 35% (16/46) strongly agreed and 59% (27/46) agreed that it would be advisable and beneficial for their organization to make use of third-party health app quality assessments in the next 2 years, while 7% (3/46) strongly disagreed with this statement.

Finally, 80% (37/46) of the health system representatives indicated that, in their opinion, it would be preferable if it was *mandatory* for health app developers to display their app’s quality assessment results, or a statement that their app has *not* been assessed, in app stores and marketing materials, while the remaining 20% (9/46) thought that such reporting should be *voluntary*.

Separate response summaries for health care institution employees and representatives of other organization types can be found in Table S7 in Multimedia Appendix 1.

#### Ranking of Assessment Options

Examining the mean rankings (from 1=most preferred to 5=least preferred) of different ways to engage with health app quality assessments revealed that, on average, health system representatives’ most preferred option was to have assessments conducted in-house (mean 2.04, SD 1.10), followed by paying for third-party assessment (mean 2.39, SD 0.98) or access to a library with assessed apps (mean 2.70, SD 1.38). Relying on developer self-assessments (mean 3.35, SD 1.06) or not making use of assessment at all (mean 4.52, SD 1.07) were, on average, the least preferred options. The same pattern of preferences was observed for both health care institution employees and other representatives when the rankings of these groups were considered separately. A chi-square test across all health system representatives indicated that the rankings were not random (*χ*^2^_4_=70.2; *P*<.001). The number of times each option received a given rank is displayed in Table 2.

**Table 2:** Indication of the frequency with which assessment options were given each rank by health system representatives, with rank 1 indicating the most preferred option and rank 5 the least preferred option (N=46).

Assessment options	Ranks, n (%)				
	1	2	3	4	5
Paid third-party assessment	9 (20)	16 (35)^a^	16 (35)^a^	4 (9)	1 (2)
Paid access to a library with assessed apps	11 (24)	13 (28)^a^	7 (15)	9 (20)	6 (13)
In-house assessment	20 (43)^a^	10 (22)	10 (22)	6 (13)	0 (0)
Developer self-assessment	4 (9)	5 (11)	11 (24)	23 (50)^a^	3 (7)
Not making use of assessment	2 (4)	2 (4)	2 (4)	4 (9)	36 (78)^a^

^a^Highest ranking frequency for each option.

#### Model Estimation Results and Relative Attribute Importance

The mixed logit model estimation results for the health system representative DCE are shown in Table 3. All attributes made a significant contribution to health system representatives’ choices, with coefficients associated with levels 2 and 3 differing significantly from those associated with the reference level 1 in all cases. All attribute levels had the expected sign and order, with health system representatives, on average, preferring assessments that have a lower cost (purchase price); make results available more quickly (ie, have a shorter assessment completion time); offer reassessment after substantial updates, rather than every year or every 2 years; are recommended by a higher proportion of peers; and lead to a larger increase in willingness to use assessed apps.

**Table 3:** Mixed logit model estimation results for health system representatives.

			Mean coefficient (SE)		*P* value		SD (SE)			*P* value
Cost (per €100)			−0.03 (0.01)		<.001		—^a^			—
Assessment completion time (per mo)			−0.16 (0.03)		<.001		—			—
**Reassessment frequency**										
	After substantial app updates (reference)	0.00 (N/A^b^)		—		—			—	
	Every y	−0.33 (0.17)		.04		0.06 (0.24)			.81	
	Every 2 y	−0.44 (0.21)		.04		0.66 (0.20)			<.001	
**Peers recommending the assessment (%)**										
	50 (reference)	0.00 (N/A)		—		—			—	
	70	0.79 (0.25)		.002		0.58 (0.51)			.25	
	90	0.82 (0.18)		<.001		0.68 (0.26)			.01	
**Increase in willingness to use assessed apps**										
	Small (reference)	0.00 (N/A)		—		—			—	
	Moderate	1.22 (0.22)		<.001		0.39 (0.52)			.46	
	Large	2.14 (0.39)		<.001		1.12 (0.42)			.01	
Alternative specific constant for “no assessment”			−1.56 (1.18)		.19		2.49 (0.59)			<.001

^a^Details for the SD (SE and *P* value) are not applicable to fixed effects.

^b^N/A: not applicable (SD, SE, and *P* values are not applicable to reference levels).

The estimated coefficients for the sensitivity analysis, excluding participants who did not select the expected option in the rationality test, are presented in Table S8 in Multimedia Appendix 1.

Relative attribute importance values indicated that increased willingness to use assessed apps had the largest impact on health system representatives’ choices (39.3%), followed by cost (22.3%). Assessment completion time (15.1%) and the percentage of peers recommending the assessment (15.1%) made a moderate contribution to choices, while reassessment frequency had the smallest impact (8.1%; refer to Figure S2 in Multimedia Appendix 1).

#### Marginal Willingness to Pay and Marginal Rates of Substitution for Assessment Completion Time

**Figure 4:**
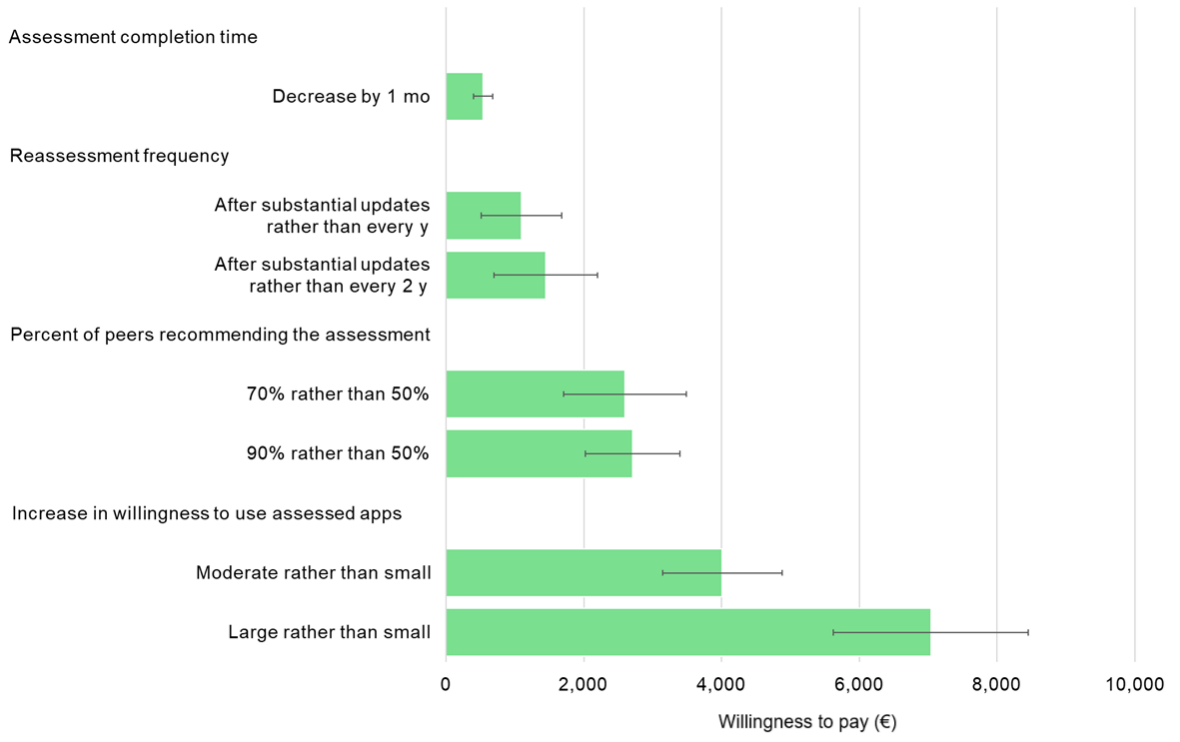
Health system representatives’ marginal willingness to pay for different assessment attribute levels (error bars represent SEs). Note that the currency exchange rate at the time of data collection was approximately €1=US $1.11.

On average, health system representatives were willing to pay an additional €7037 (95% CI €4267-€9806) if an assessment yields a large, rather than a small, increase in willingness to use assessed apps; and €2709 more (95% CI €1355-€4063) if the assessment is recommended by 90%, rather than 50%, of peers. Marginal willingness to pay for app reassessment after substantial updates, rather than every 2 years, was €1448 (95% CI −€25 to €2921); and a decrease in assessment completion time of 1 month was, on average, worth €542 (95% CI €269-€816) to health system representatives (refer to Figure 4 and Table S9 in Multimedia Appendix 1 for the full results).

Furthermore, health system representatives were willing to wait an additional 13 months (95% CI 7-19 mo) for the assessment results if the assessment is associated with a large, rather than a small, increase in willingness to use assessed apps; and a further 5 months (95% CI 2-8 mo) if the assessment is recommended by 90%, rather than 50%, of peers. For a cost reduction of €100, health system representatives were willing to wait an additional week (0.2 mo; 95% CI 0.1-0.3 mo; refer to Table S10 in Multimedia Appendix 1 for the full results).

#### Predicted Assessment Uptake

The predicted assessment uptake was examined for the best-, middle-, and worst-case scenarios, with all attribute levels set to the most preferred, middle, and least preferred levels, respectively.

For the best-case scenario, the predicted assessment uptake among health system representatives was 91.1% (95% CI 68%-99.2%), while for the middle- and worst-case scenarios, the predicted uptake was 78.7% (95% CI 48.7%-94.2%) and 35% (95% CI 11.8%-65.8%), respectively.

## Discussion

### Principal Findings

The aim of this study was to examine preferences and willingness to pay for health app assessments with different value propositions among potential customers of such assessments, namely health app developers and health system representatives.

The DCEs revealed that, among health app developers, the attributes with the largest impact on assessment choices were clinical care uptake (guideline integration and reimbursement or procurement) and cost (purchase price). Increased willingness to use assessed apps and app store integration had a moderate impact on choices, and assessment completion time and developer time investment made the smallest contribution. Among health system representatives, increased willingness to use assessed apps had the largest impact on participants’ choices, followed by cost. Assessment completion time and the percentage of peers recommending the assessment made a moderate contribution to choices, and reassessment frequency had the smallest impact.

On average, health app developers were willing to pay an additional €9020 (95% CI €4968-€13,072) if an assessment facilitates guideline integration and procurement or reimbursement. The marginal willingness to pay was only approximately a third of this amount if an assessment offers full app store integration (assessment score display and impact on app search result rankings; €3052, 95% CI €1722-€4381); or leads to a large, rather than a small, increase in willingness to use assessed apps (€3090; 95% CI €1341-€4840). Health system representatives were, on average, willing to pay €7037 (95% CI €4267-€9806) more if an assessment results in a large, rather than a small, increase in willingness to use assessed apps; and an additional €2709 (95% CI €1355-€4063) if the assessment is recommended by 90%, rather than 50%, of peers.

### Attitudes and Preferences Regarding Health App Assessment Attributes

The interest in, and predicted uptake of, health app assessments was high among both health app developers and health system representatives. In both groups, a majority of the participants (health app developers: 38/41, 93%; health system representatives: 43/46, 93%) agreed that it would be advisable and beneficial for their organization to make use of third-party app assessments in the next 2 years. The predicted uptake of assessments that offered the preferred values for all attributes was 88.6% among app developers and 91.1% among health system representatives. This indicates that both groups are viable target customers for health app assessments if their expectations regarding cost and benefits are met.

In this context, it is worth noting that health system representatives’ most preferred option for engaging with app assessments was to have apps evaluated in-house. This may partly be the case because approximately half of the participants (21/46, 46%) in this group worked at large or very large organizations that may have their own product evaluation processes in place. For instance, large health care institutions may contain procurement or IT departments that routinely evaluate products, including digital health technologies. Where such a trusted evaluation process is available in-house, this may be the routine approach for assessing health care products in general and, as such, also the preferred approach for ensuring the quality of used health apps. Nevertheless, making use of third-party assessment was, on average, the second most favored option. Together with the DCE results, this suggests a solid interest in app assessments from health system organizations, as long as a suitable cost-benefit balance is offered.

Specifically, the DCE results indicate that, to maximize uptake among health system organizations, it is important to demonstrate that an assessment substantially increases the public’s and health care professionals’ willingness to use health apps. This impact could, for instance, be demonstrated by conducting a controlled study in which participants are presented with hypothetical apps that either carry or do not carry a quality assessment label and are asked whether they would use or recommend these apps. A recent study using this approach showed that health care professionals’ willingness to recommend health apps was significantly higher for apps that carried a quality label than for those that did not [47]. Besides this direct effect of a quality label, there may also be a more indirect impact of assessment on health care professionals’ willingness to use evaluated apps *if* the assessment enables the integration of health apps into clinical guidelines. This may further encourage health app use and recommendation, given that health care professionals rely on clinical guidelines to inform their practice [48,49].

Similarly, assessment may also increase the use of health apps among the public. Yet-to-be-published research from the Label2Enable project shows that nearly 9 in 10 surveyed European citizens think that the government should review and rate apps or pay another organization to do so to help them choose a health app. This indicates a high demand for guidance regarding health app choices among the public. In the future, this research could be extended to further quantify the increase in willingness to use health apps as a result of trust in and visibility, transparency, and clarity of different quality assessments.

Our findings further show that enabling or facilitating the clinical guideline integration and reimbursement or procurement of assessed apps is an important factor for maximizing assessment uptake among health app developers. This requires the close collaboration of assessment providers with health authorities, health care institutions, and health insurers. While reimbursement of health apps is arguably still in its early stages, reimbursement mechanisms have started to emerge across Europe, including through the German digital health app assessment [50], the Belgian National Institute for Health and Disability Insurance assessment [51], and the French “early access to reimbursement for digital devices” program [52]. Our findings underscore that, as expected, such reimbursement programs provide a substantial incentive for developers to submit their apps for associated quality assessments. However, even if reimbursement is offered, assessment scaling can be a challenge [48,53]. Therefore, it is important to also optimize other assessment characteristics, such as cost, developer time investment, and assessment completion time, as well as the visibility of and trust in the assessment. In addition, further efforts are needed to encourage professional medical societies and other relevant bodies to include health apps in clinical recommendations and guidelines [48,49].

Another attribute with a moderate impact on developers’ assessment choices was app store integration. Notably, such integration has substantial benefits beyond being an incentive for app developers to opt for quality assessment. Currently, search result rankings in major app stores are influenced by download numbers and user ratings, among other factors [54]. However, research has shown that these metrics are not significantly correlated with health app quality, as determined by in-depth assessment [55]. Given that users rarely consider more than the first 5 to 10 apps listed in the search results before making a choice [56], the current ranking system may indirectly encourage the use of potentially ineffective or unsafe health apps. This could be partially counteracted by incorporating trusted third-party quality assessment scores into search result ranking algorithms, ideally placing a higher weight on such scores than on download numbers and user ratings.

A further attribute that warrants consideration beyond its influence on assessment choices is reassessment frequency. Perhaps somewhat surprisingly, this attribute had the smallest relative impact on health system representatives’ assessment choices. A potential contributing factor to this finding may have been uncertainty regarding how frequently reassessment would take place if it was contingent on substantial app updates that affect privacy or health-related risks, which was the reference category for the DCE analysis. It was not possible to specify the reassessment frequency for this scenario because this likely varies widely across apps. Recent data show that the median time since the last update among the highest-ranking health and fitness apps in the Google Play Store is just 14 days [57]. However, updates that impact evaluated components to an extent that warrants reassessment are likely to be substantially less frequent. Nevertheless, due to the fast pace of app development, reassessment at regular intervals or after substantial app changes is important to ensure that key quality information is applicable to the most recent version of the app. If no reassessment takes place or if it is too infrequent or poorly timed, the result could be reliance on outdated information. This, in turn, could potentially lead to the recommendation of health apps that no longer meet quality standards, which could undermine trust in the assessment. Future quantitative and qualitative research could further examine how frequently health apps, on average, provide updates that affect privacy and health risks and to what extent the frequency of such updates may affect preferences for reassessment, as well as trust in assessment results, among health system representatives and app users.

### Willingness to Pay for Health App Assessments

The findings of this study provide useful insights regarding the willingness to pay for health app assessments with varying characteristics across potential customer groups. For instance, the DCE results indicate that health app developers would be willing to pay €6843 more for an assessment with the middle attribute values *compared to an assessment with the least preferred* values, that is, for an assessment requiring 20, rather than 40, hours of time investment (€1076-€1028 = €48); providing assessment results in 3, rather than 6, months (€425 × 3 = €1275); facilitating clinical guideline integration for high-scoring apps (without reimbursement; €3332); displaying assessment results in app stores (without impact on search result ranking; €1240); and resulting in a moderate, rather than a small, increase in willingness to use assessed apps (€948). Similarly, health system representatives would be willing to pay €8587 more for a “middle case” assessment *compared to one with the least preferred attribute values*, that is, for an assessment that provides results in 3, rather than 6, months (€542 × 3 = €1626); is recommended by 70%, rather than 50%, of their peers (€2600); offers reassessment every year, rather than every 2 years (€1448-€1098 = €350); and leads to a moderate, rather than a small, increase in willingness to use assessed apps (€4011).

In this context, it should be noted that the DCEs used in this study only investigated willingness to pay for one-off assessments to avoid dependencies between attributes, such as between cost, time investment, and reassessment frequency. For this reason, the health system DCE instructed participants to assume that reassessment costs would not be covered by their organization. Therefore, a further examination of how reassessment frequency affects willingness to pay is warranted. This is especially important given the fast pace of digital health development, as mentioned previously, and considering the additional cost and resource requirements for assessment organizations that are implementing frequent reassessment.

Moreover, it should be taken into account that the willingness to pay estimates for app developers may not fully capture the necessary trade-offs in resource allocation, especially when it comes to meeting mandatory versus voluntary requirements. Specifically, ensuring that a health app meets regulatory requirements, such as those outlined by the General Data Protection Regulation and MDR, necessitates resource investment; and the more time and money have already been spent on aligning a given app to these mandatory requirements, the less willing or able developers may be to invest additional resources in voluntary quality assessment. This point was raised by several participants in a free-text comment section at the end of the current survey (eg, “We have already spent upwards of 200,000 Euros obtaining our MDR certification - why should we spend more?”). To address these concerns, it is advisable to not only emphasize the additional benefits offered by a given voluntary assessment but also allow for the reuse of documentation from mandatory certifications, where appropriate. This helps to minimize resource requirements and avoid duplicating the MDR related evaluation performed by notified bodies.

Relatedly, it should also be considered how resource requirements, including for covering assessment fees, affect smaller versus larger organizations. As part of this study, both assessment organization interviewees and survey participants voiced concerns that assessment costs or extensive time requirements for app developers could disadvantage smaller companies that do not have the necessary resources to pay for or invest time in the assessment process. This could negatively impact innovation by creating additional barriers for the scaling of start-ups, especially if assessment outcomes, or the lack thereof, have a substantial impact on users’ health app choices. Some interviewees suggested that adjusting assessment fees based on company size or charging health system organizations instead of app developers may partially address this issue.

### Next Steps

As mentioned in the Introduction section, a major challenge for current health app assessment initiatives, including national programs, is reaching a scale at which they can evaluate a substantial proportion of the large number of health apps on the market [29,31,32]. Such scaling requires both an efficient assessment process with a sufficient number of assessors and a value proposition and pricing that make the assessment attractive to a substantial number of customers, such as health app developers or health system organizations.

This study is part of a larger initiative that aims to establish such a scalable assessment program across Europe, using the quality requirements of CEN ISO/TS 82304-2. In terms of the organizational implementation of this program, it is envisioned that there will be a central governing body, alongside a distributed network of assessment organizations located across Europe, that will carry out the health app evaluations. The intention is to rely, in the first instance, on organizations that already provide digital health assessment services (using various evaluation frameworks) and, therefore, have the necessary resources and structures in place. A pilot study was recently carried out with 5 such organizations to examine their interrater reliability for a draft version of the CEN ISO/TS 82304-2 assessment, which yielded promising results [58].

To enhance the efficiency, quality, affordability, and scalability of CEN ISO/TS 82304-2 assessments and reassessments as well as to avoid a duplication of assessment efforts, automated evaluation will be used, where feasible, and the results from trusted existing assessments will be accepted as evidence for subsets of CEN ISO/TS 82304-2 quality requirements. It is expected that the CEN ISO/TS 82304-2 assessment will be comprehensive enough to provide health care decision makers with the vast majority of the information they require, reducing their assessment efforts to merely examining a small number of context-specific requirements. This expectation is supported by a recent, comparative analysis by the Catalan government body responsible for health app evaluations, which showed that the CEN ISO/TS 82304-2 app assessment handbook covers 93% of the evaluation criteria considered important by the Catalan assessment body [59].

The findings of this study will be used to inform decisions regarding the pricing and value proposition of the CEN ISO/TS 82304-2 assessment. The study showed that both health app developers and health system representatives widely agree that their organizations would benefit from health app assessments and that both groups demonstrate a sufficient willingness to pay for such assessments if their preferences are met. Therefore, both health app companies and health system organizations will likely be targeted as customers of the CEN ISO/TS 82304-2 assessment. In addition, it will be explored how the preferences expressed by these stakeholders as part of this study can be realized to ensure optimal assessment uptake.

The hope is that through the efficient, distributed implementation of the app evaluation and the alignment to customer preferences, the CEN ISO/TS 82304-2 assessment program will eventually be able to provide evaluation results for a substantial proportion of health apps available on the market while using a thorough and consistent assessment approach.

### Limitations

A few limitations of this study should be noted. First, participants who took the time to complete the DCE survey were likely already interested in or well informed about the topic of health app assessment. Indeed, this study’s results indicate that the majority of participants had previous experience with, and a positive attitude toward, health app assessment. Therefore, the recruited sample may not be entirely representative of all health app developers or health system representatives across Europe. It is possible that willingness to pay and assessment uptake may, in reality, be lower than indicated by these findings (eg, due to a greater negative effect of assessment cost or more opt-out decisions in the larger population compared to this study’s sample). Moreover, it is possible that other attributes that were not included in this study may be of importance to some stakeholders, the inclusion of which could result in differing preferences and willingness to pay values. Future research could examine this further by actively recruiting participants with less awareness of, and less positive attitudes toward, digital health and quality assessments.

Second, due to the limited sample size, we were not able to perform subgroup analyses for participants with different characteristics (eg, those with previous positive, negative, or no assessment experience) or for different organization types (eg, for health care institutions, authorities, insurers, or health technology assessment bodies; or for health app developers with varying business models). Future research is warranted to examine whether preferences and willingness to pay for health app assessments differ across these subgroups.

Finally, when interpreting the study findings, it should be kept in mind that the estimates for health app developers and health system representatives cannot be directly compared. This is partly due to the use of different attributes within the DCEs, which was necessary to account for the varying relevance of different attributes across the 2 groups (refer to the Methods section for details).

### Conclusions

This study aimed to examine preferences and willingness to pay for health app assessments with different value propositions among potential customers of such assessments. Among both app developers and health system representatives, interest in, and the predicted uptake of, health app assessments was high if the most preferred assessment attribute values were offered. Moreover, for health app developers and health system representatives, the attributes with the largest impact on assessment choices were facilitation of clinical care uptake (guideline integration and reimbursement or procurement) and increased willingness to use assessed apps, respectively. The study’s findings provide valuable information, including marginal willingness to pay estimates, that can be used to inform decision-making and support the scaling of health app assessments.

## Data Availability

The datasets generated and analyzed during this study are available from the corresponding author on reasonable request.

## Appendix

Multimedia Appendix 1 Supplementary methods and results.

## Abbreviations

CE: Conformité Européenne
DCE: discrete choice experiment
MDR: Medical Device Regulation
