# Supplementary material for: Preferences and Willingness to Pay for Health App Assessments Among Health Care Stakeholders: Discrete Choice Experiment
Source: JMIR Mhealth Uhealth. 2025 May 26;13:e57474. doi: 10.2196/57474 (PMC12149772; doi:10.2196/57474)
Supplement: Multimedia Appendix 1 [file mhealth_v13i1e57474_app1.pdf]

Supplementary materials

Table S1: Background information for assessment organizations included in the interviews

**Assessment name**	**Affiliated organization(s)**	**Country (region)**	**Website link**
mHealthBelgium	Joint initiative between the Belgian Federal Government and the technology industry	Belgium	https://mhealthbelgium.be/validation-pyramid
Digi-HTA	Finnish Coordinating Centre for Health Technology Assessment (FinCCHTA) and the University of Oulu	Finland	https://oys.fi/fincchta/digi-hta/about-digi-hta/
TICSS Guarantee Certification	TIC Salut Social Foundation, an entity of the Catalan Department of Health	Spain (Catalonia)	https://ticsalutsocial.cat/en/projecte/mhealth/
GGD AppStore	The Association of Regional Public Health Services (GGD) and Regional Medical Emergency Preparedness and Planning Services (GHOR)	Netherlands	https://www.ggdappstore.nl/Appstore/Testmethode
MindApps.dk	Centre for Digital Psychiatry, Southern Denmark	Denmark (Southern Denmark)	https://mindapps.dk
Helsedirektoratet 'Safer health apps'	The Directorate of Health, Norwegian Health Network, and the Directorate for e-Health	Norway	https://www.helsedirektoratet.no/om-oss/forsoksordninger-og-prosjekter/tryggere-helseapper
Digital Technology Assessment Criteria for health and social care (DTAC)	National Health Service (NHS) England	England	https://transform.england.nhs.uk/key-tools-and-info/digital-technology-assessment-criteria-dtac/

Table S2: Countries in which health app developers were located

**Country**	**Frequency, n**	**Percentage**
Bulgaria	1	2.5
Croatia	1	2.5
Denmark	3	7.5
France	8	20
Germany	4	10
Greece	1	2.5
Ireland	1	2.5
Italy	1	2.5
Montenegro	1	2.5
Netherlands	4	10
Norway	1	2.5
Portugal	3	7.5
Romania	1	2.5
Slovenia	1	2.5
Spain	2	5
Sweden	2	5
Switzerland	1	2.5
United Kingdom	4	10

Table S3: Mixed logit model estimation results for health app developers excluding participants who did not respond as expected in the rationality test (N excluded = 3)

**Attribute/ level**	**Mean coefficient**	**SE**	***P*-value**	**SD**	**SE**	***P*-value**
**Cost** (per €100)	-0.03	0.01	<.001	—[a]	—	—
**Assessment completion time** (per month)	-0.15	0.03	<.001	—[a]	—	—
**Developer time investment**						
10 hours (reference)	0	—[b]	—	—	—	—
20 hours	-0.29	0.24	.23	0.47	0.35	.18
40 hours	-0.42	0.20	.04	0.73	0.28	.01
**Impact on clinical care uptake**						
None (reference)	0	—[b]	—	—	—	—
Guideline integration	1.34	0.26	<.001	0.80	0.28	<.001
Guideline integration & procurement or reimbursement	3.64	0.62	<.001	2.26	0.35	<.001
**App store integration**						
None (reference)	0	—[b]	—	—	—	—
Assessment score display	0.44	0.17	.008	0.10	0.09	.27
Assessment score display & impact on app search result rankings	1.26	0.21	<.001	0.51	0.20	.01
**Increase in willingness to use assessed apps**						
Small (reference)	0	—[b]	—	—	—	—
Moderate	0.49	0.24	.04	0.49	0.28	.08
Large	1.22	0.40	.002	0.87	0.33	.01
**Alternative specific constant** for 'no assessment'	0.72	0.61	.24	1.93	0.36	<.001

[a] Details for the SD (SE and p-value) are not applicable to fixed effects.
[b] SD, SE, and p-values are not applicable to reference levels.

Figure S1: Relative attribute importance for health app developers (error bars represent SEs).

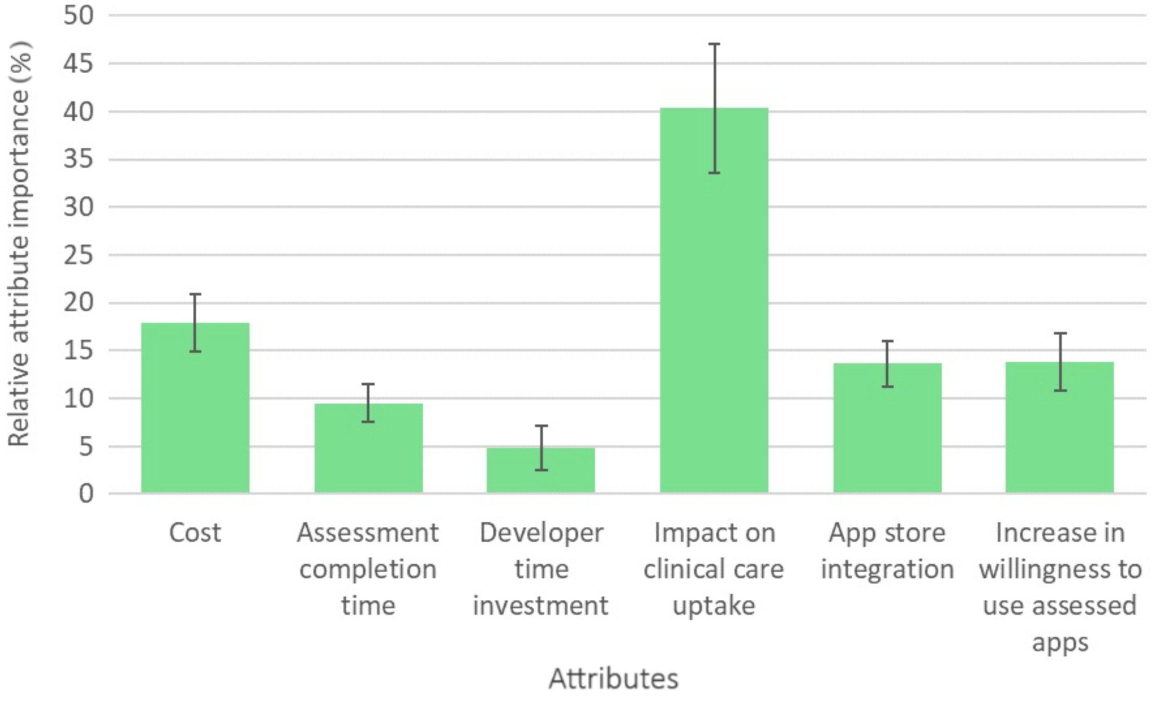

Table S4: Marginal willingness to pay among health app developers

	Marginal willingness to pay (in Euros)			
	Mean	SE	95% Confidence Interval	
			Lower limit	Upper limit
**Assessment completion time**				
Decrease by 1 month	425	117	196	654
**Developer time investment**				
10 rather than 20 hours	1,028	624	-194	2,250
10 rather than 40 hours	1,076	542	14	2,137
**Impact on clinical care uptake**				
Guideline integration	3,332	681	1,997	4,667
Guideline integration & procurement or reimbursement	9,020	2,068	4,968	13,072
**App store integration**				
Display of assessment scores	1,240	439	379	2,101
Display of assessment scores & impact on search result ranking	3,052	678	1,722	4,381
**Increase in willingness to use assessed apps**				
Moderate rather than small	948	739	-501	2,396
Large rather than small	3,090	893	1,341	4,840

Table S5: Marginal rates of substitution (MRS) for assessment completion time among health app developers

	MRS for assessment completion time (in months)			
	Mean	SE	95% Confidence Interval	
			Lower limit	Upper limit
**Cost**				
Decrease by €100	0.2	0.1	0.1	0.4
**Developer time investment**				
10 rather than 20 hours	2.4	1.5	-0.5	5.3
10 rather than 40 hours	2.5	1.3	0.0	5.0
**Impact on clinical care uptake**				
Guideline integration	7.8	2.3	3.4	12.3
Guideline integration & procurement or reimbursement	21.2	6.7	8.1	34.4
**App store integration**				
Display of assessment scores	2.9	1.1	0.7	5.1
Display of assessment scores & impact on search result ranking	7.2	2.0	3.2	11.1
**Increase in willingness to use assessed apps**				
Moderate rather than small	2.2	1.9	-1.5	6.0
Large rather than small	7.3	2.5	2.4	12.1

Table S6: Countries in which health system representatives were located

Country	Frequency, n	Percentage
Belgium	5	10.9
Croatia	4	8.7
France	2	4.3
Ireland	2	4.3
Italy	1	2.2
Lithuania	3	6.5
Netherlands	5	10.9
Norway	1	2.2
Portugal	2	4.3
Spain	14	30.4
Switzerland	1	2.2
Ukraine	1	2.2
United Kingdom	5	10.9

Table S7: Summary of experiences with and attitudes towards health app assessment reported by health system representatives from different types of organizations

	**All health system representatives (n/N)**	**Employees of healthcare institutions (n/N)**	**Employees of health authorities, insurers and HTA bodies (n/N)**
**Had previous experience with health app assessment**	73.9% (34/46)	82.1% (23/28)	61.1% (11/18)
**Evaluation of previous experience** with health app assessment (if any)			
Mostly positive	76.5% (26/34)	69.6% (16/23)	90.9% (10/11)
Neutral	20.6% (7/34)	26.1% (6/23)	9.1% (1/11)
Mostly negative	2.9% (1/34)	4.3% (1/23)	0% (0/11)
Agreement regarding whether it would be advisable and **beneficial for their organization to make use of third-party health app quality assessments** in the next 2 years			
Strongly agree	34.8% (16/46)	32.1% (9/28)	38.9% (7/18)
Agree	58.7% (27/46)	67.9% (19/28)	44.4% (8/18)
Disagree	0% (0/46)	0% (0/28)	0% (0/18)
Strongly disagree	6.5% (3/46)	0% (0/28)	16.7% (3/18)
Were of the opinion that it would be **preferable if it was *mandatory*** (rather than voluntary) **for health app developers to display their app's quality assessment results**, or a statement that their app has *not* been assessed, in app stores and marketing materials	80.4% (37/46)	82.1% (23/28)	77.8% (14/18)

HTA; Health technology assessment

Table S8: Mixed logit model estimation results for health system representatives excluding participants who did not respond as expected in the rationality test (N excluded = 5)

Attribute/ level	Mean coefficient	SE	*P*-value	SD	SE	*P*-value
**Cost** (per €100)	-0.04	0.01	<.001	—[a]	—	—
**Assessment completion time** (per month)	-0.21	0.04	<.001	—[a]	—	—
**Reassessment frequency**						
After substantial updates (reference)	0	—[b]	—	—	—	—
Every 1 year	-0.35	0.19	.06	0.28	0.29	.33
Every 2 years	-0.42	0.24	.09	0.82	0.26	<.001
**Percent of peers recommending the assessment**						
50% (reference)	0	—[b]	—	—	—	—
70%	0.55	0.25	.03	0.02	0.17	.89
90%	1.01	0.23	<.001	0.55	0.21	.01
**Increase in willingness to use assessed apps**						
Small (reference)	0	—[b]	—	—	—	—
Moderate	1.40	0.26	<.001	0.60	0.33	.07
Large	2.58	0.45	<.001	1.26	0.28	<.001
**Alternative specific constant** for 'no assessment'	-1.64	0.62	.008	2.49	0.50	<.001

[a] Details for the SD (SE and p-value) are not applicable to fixed effects.
[b] SD, SE, and p-values are not applicable to reference levels.

Figure S2: Relative attribute importance for health system representatives (error bars represent SEs).

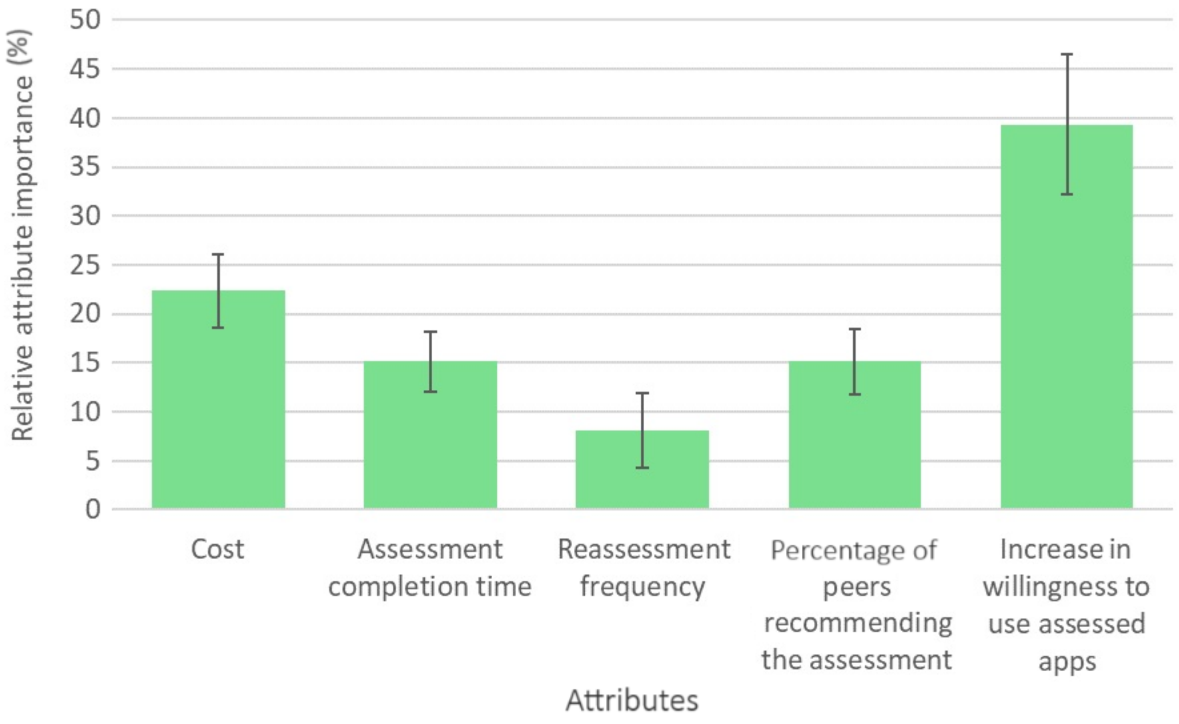

Table S9: Marginal willingness to pay among health system representatives

	Marginal willingness to pay (in Euros)			
	**Mean**	**SE**	**95% Confidence Interval**	
			**Lower limit**	**Upper limit**
**Assessment completion time**				
Decrease by 1 month	542	140	269	816
**Reassessment frequency**				
After substantial updates rather than every 1 year	1,098	585	-49	2,246
After substantial updates rather than every 2 years	1,448	751	-25	2,921
**Percent of peers recommending the assessment**				
70% rather than 50%	2,600	889	858	4,342
90% rather than 50%	2,709	691	1,355	4,063
**Increase in willingness to use assessed apps**				
Moderate rather than small	4,011	865	2,315	5,706
Large rather than small	7,037	1,413	4,267	9,806

Table S10: Marginal rates of substitution (MRS) for assessment completion time among health system representatives

	MRS for assessment completion time (in months)			
	Mean	SE	95% Confidence Interval	
			Lower limit	Upper limit
**Cost**				
Decrease by €100	0.2	0.05	0.1	0.3
**Reassessment frequency**				
After substantial updates rather than every 1 year	2.0	0.9	0.3	3.8
After substantial updates rather than every 2 years	2.7	1.2	0.3	5.0
**Percent of peers recommending the assessment**				
70% rather than 50%	4.8	1.8	1.4	8.2
90% rather than 50%	5.0	1.4	2.3	7.7
**Increase in willingness to use assessed apps**				
Moderate rather than small	7.4	1.7	4.1	10.7
Large rather than small	13.0	3.1	7.0	19.0

Analysis of opt-out decisions

On average, participants from health app development companies opted for 'no assessment' on 27.8% (SD = 25.4) of trials, while health system representatives choose 'no assessment' on 19.3% (SD = 26.7) of trials.

Linear regression analyses were conducted to examine whether responses to the background questions predicted the percent of times participants opted for 'no assessment'. The analyses were conducted separately for health app developers and health system representatives, given that these groups were presented with different scenarios as part of the DCE, and thus their opt-out decisions may have been driven by different factors. The following variables were entered as predictors into the regression model: organization size (dummy coded; micro [reference], small, medium, large, very large), organization type (dummy coded and included for health system representatives only; public healthcare organization [reference], private healthcare organization, health authority, public health insurer, private health insurer, and health technology assessment body), purchasing power parities of the country in which the participants' organization was located (as of 2022, see [1]), previous assessment experience (dummy coded; not applicable [reference], mostly negative, neutral, mostly positive), and attitude towards assessment (as indicated by a four-point Likert scale rating of how strongly participants agreed or disagreed that making use of health app quality assessment in the next 2 years would be beneficial and advisable for their organization).

This model did not reached significance for either health system representatives ($r^2 = 0.26$, $F(14,28) = 0.71$, $P = .75$) or for health app developers ($r^2 = 0.27$, $F(9,29) = 1.21$, $P = .33$).

References

1. OECD. Purchasing power parities (PPP). 2022. Available from: https://data.oecd.org/conversion/purchasing-power-parities-ppp.htm [accessed Oct 24, 2023]
